# FDTD Analysis of End-Fire Coupling Between Mid-IR On-Chip Light Sources on Si and As_2_Se_3_-on-CaF_2_ Waveguide from 3 to 8 µm for Spectroscopic Sensing

**DOI:** 10.3390/s26206632

**Published:** 2026-10-20

**Authors:** Saranisorn Srikam, Pichet Limsuwan, Papichaya Chaisakul

**Affiliations:** 1 Department of Physics, Faculty of Science, Kasetsart University, Bangkok 10900, Thailand; saranisorn.s@ku.th; 2 Department of Physics, Faculty of Science, King Mongkut’s Institute of Technology Ladkrabang, Bangkok 10520, Thailand; pichet.lim@kmutt.ac.th; 3 Department of Physics, Faculty of Science, King Mongkut’s University of Technology Thonburi, Bangkok 10140, Thailand

**Keywords:** Mid-IR light sources on Si, As_2_Se_3_-on-CaF_2_ waveguide, chip-scale optical integration

## Abstract

This work demonstrates that the As_2_Se_3_-on-CaF_2_ waveguide can enable optical integration with three distinct mid-infrared (mid-IR) light sources on silicon emitting at wavelengths of 3.6, 4.8, and 7.4 μm, while sustaining a competitive waveguide-based sensing figure of merit within a mid-IR spectral range of 3–8 µm. Despite considerable variations in lasing optical mode sizes and polarizations from three different laser chips, the As_2_Se_3_-on-CaF_2_ waveguide can facilitate efficient butt-coupling with each source while maintaining the same As_2_Se_3_ waveguide thickness. This permits the implementation of As_2_Se_3_-on-CaF_2_ waveguides, in which the waveguide width can be tailored to achieve both competitive optical coupling performance with the light sources and a high sensing figure of merit across the entire wavelength range of interest. This work demonstrates the attractiveness of the chalcogenide As_2_Se_3_-on-CaF_2_ waveguide in terms of design flexibility for chip-scale optical integration in the important mid-IR wavelength ranges.

## 1. Introduction

Gas detection plays an important role in modern life. Gases are measured in many applications such as human and environmental care and industrial and agricultural emissions [1,2,3,4,5,6,7,8,9,10,11,12]. Interestingly, gas sensors developed with optical chip technology have increasingly received significant attention based on several configurations, including a plasmonic nanocavity [13], Mach–Zehnder interferometer [14], photonic crystal microcavity [15], microring resonator [16,17], and metasurface [18]. Nevertheless, optical sensing relying on refractive index changes according to the surrounding molecules normally lacks chemical specificity without additional surface-functionalization techniques [19]. Promisingly, the optical detection of gas molecules using spectroscopic techniques of fingerprint absorption in the mid-infrared (mid-IR) region can enable fast detection with excellent selectivity according to the unique absorption features of each gas molecule [20,21]. Accordingly, numerous theoretical and experimental studies have investigated on-chip optical sensors based on the absorption of gas molecules in the mid-infrared region using a micron-scale optical waveguide platform [22,23,24,25,26,27,28,29], exploiting the interaction between the evanescent field around the waveguide and gas molecules. Previous on-chip mid-IR gas sensing theoretical and experimental works have explored Si-on-sapphire [30], Si_3_N_4_ [28,31], Si [32,33,34,35,36,37,38,39,40], and chalcogenide [41,42,43,44] waveguides mainly for narrowband sensing around single gas absorption lines. Moreover, Si and Si_3_N_4_ platforms cannot cover a full 3–8 μm window [45,46] due to material transparency limits [47,48,49], and although Ge-rich SiGe-on-graded-buffer waveguides can span the full 3–8 μm range [50,51], their low index contrast limits the evanescent field needed for sensitive detection [52].

Moreover, it is important to note that, although all of the aforementioned studies impressively investigated the sensing potential of chip-scale waveguide structures, the integration of a light source into the sensing chip was not taken into account, and external benchtop commercial lasers were typically employed [33,34,35,36,37,38,39,40,41,42,43,44]. Prior inter-chip optical integration studies focused on optical integration over a narrow wavelength range from near-IR telecom applications using III-V DFB laser-to-Si_3_N_4_ waveguide chips at ~1.55 μm [53,54,55] to short-wave IR Si-based waveguide coupling at ~2 μm [56,57]. In the mid-IR, QCL laser coupling has been demonstrated with Ge-rich SiGe-on-graded-buffer waveguides at ~7.4 μm [58], Ge-on-Si waveguides at ~7.2 μm [59] (the latter showed higher efficiency due to greater index contrast, although Ge-Si interface defects may cause scattering loss at shorter wavelengths [60,61]), and Ge-on-SOI waveguides at 5 μm [62].

As aforementioned, previous works on on-chip mid-IR gas sensing have focused mainly on narrowband sensing around single gas absorption lines, and wideband integration of a light source into the sensing chip was largely unaddressed in the mid-IR wavelength range of 3–8 µm. In this study, we aimed to explore the possibility of creating a waveguide platform that can accommodate mid-IR light sources over a wide mid-IR wavelength range from 3 to 8 µm while maintaining competitive waveguide-based sensing potentials. Via 3D finite-difference time-domain (3D-FDTD) simulation, we investigated butt-coupling chip-scale integration between an As_2_Se_3_-on-CaF_2_ waveguide chip and three different on-chip mid-IR laser sources, including an interband cascade laser (ICL) on Si [63], a quantum cascade laser (QCL) on Si [64], and a DFB-QCL laser on Si [65], emitting at wavelengths of 3.6, 4.8, and 7.4 μm, respectively. Despite significant differences in the lasing optical mode size and polarization among different lasers, we have found that the As_2_Se_3_-on-CaF_2_ waveguide platform can be employed to accommodate efficient butt-coupling with all of them, while the same As_2_Se_3_ waveguide thickness can be maintained. Significantly, this allows the system to be potentially processed on the same As_2_Se_3_-on-CaF_2_ waveguide platform, the width of which can be flexibly adapted to obtain both good optical coupling performance and competitive sensing figure of merit [35,66,67]. Studies have demonstrated the attractiveness of the chalcogenide As_2_Se_3_-on-CaF_2_ waveguide platform in terms of the design flexibility to obtain both high coupling efficiency and a high spectroscopic sensing figure of merit, enabling integration with different mid-IR light sources on Si over the mid-IR wavelength range of 3 to 8 µm. In the 3D-FDTD simulations (Ansys-Lumerical: 2025 R1), Maxwell’s wave equations were solved with a smallest mesh size of ~2 nm, and a perfectly matched layer (PML) was used to suppress reflections from the simulation region boundaries. Mesh convergence was verified prior to conducting the simulations to ensure that the results were independent of the mesh resolution. Symmetric and anti-symmetric boundary conditions were applied for quasi-TM and quasi-TE light, respectively, to reduce the computational load. A mode source was used to launch the optical mode at each corresponding wavelength, according to the specific laser waveguide structure under consideration. To measure optical transmission, two power monitors were placed along the propagation path in every case—one at the input and one at the output—enabling the transmission value along the optical path of interest to be determined.

## 2. Investigated Structures of Mid-IR Laser Sources on Si and As_2_Se_3_-on-CaF_2_ Waveguides

To initially verify the challenges and the potential of having a waveguide platform that can accommodate mid-IR light sources over a wide mid-IR wavelength range from 3 to 8 µm, the optical modes from the three laser sources with different mode sizes and polarization were investigated together with the optical modes supported by the As_2_Se_3_-on-CaF_2_ waveguide platform over the same spectral range at the same waveguide thickness.

### 2.1. Mid-IR Laser Sources on Si

**Figure 1:**
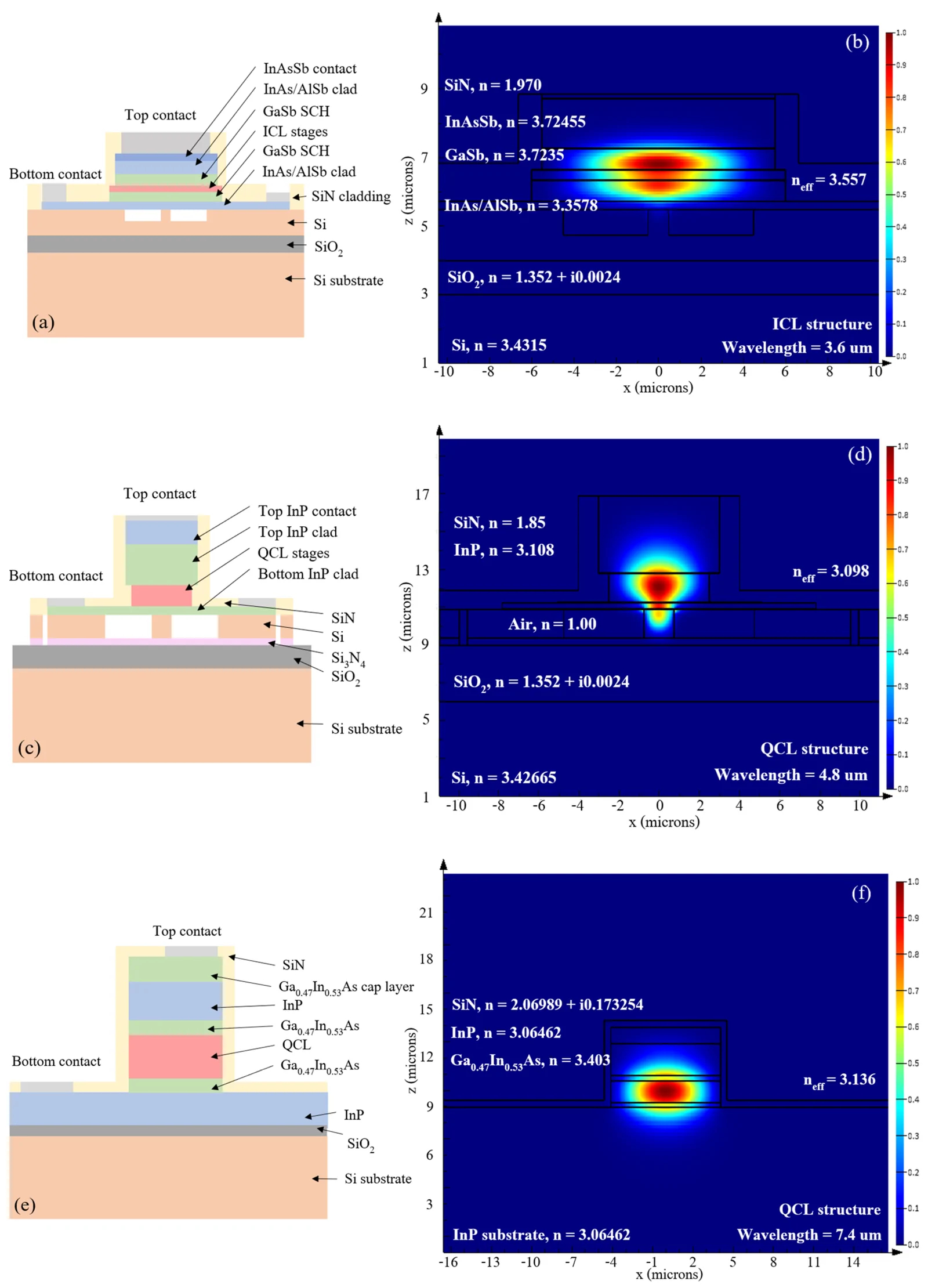
(**a**) Cross-section (x–y plane) of a reported interband cascade ICL structure on Si [63], emitting quasi-TE light around 3.6 μm with (**b**) its optical mode. (**c**) Cross-section of a reported quantum cascade laser (QCL) on Si [64], emitting quasi-TM light around 4.8 μm with (**d**) its optical mode. (**e**) Cross-section of a reported DFB-QCL laser on Si [65], emitting quasi-TM light around 7.4 μm with (**f**) its optical mode. The values of the refractive indices of different layers were deduced from [65,69,70,71,72,73].

Figure 1 shows the optical modes and the structure of the three different laser sources, in order to first observe the difference in lasing optical mode size and polarization from different mid-IR laser sources reported across optical wavelength ranges from 3 to 8 µm on Si. Figure 1a shows the cross-section of a reported interband cascade ICL structure on Si [63], emitting quasi-transverse-electric (quasi-TE) light around 3.6 μm with its optical mode as shown in Figure 1b. The structure consists of a III-V mesa coated with a SiN layer and a Si rib waveguide under the mesa with the ICL layer based on the seven-stage ICL structure [68,69]. Figure 1c shows the cross-section of a reported quantum cascade laser (QCL) on Si [64], emitting quasi-transverse-magnetic (quasi-TM) light around 4.8 μm with its optical mode as shown in Figure 1d. The refractive index values of the constituent layers were obtained from [70] for InAs, InSb, GaSb, and GaAs; [71] for AlSb; [72] for AlAs; and [65,69,73] for the detailed ICL, QCL, and DFB-QCL structures, respectively (see Supplementary File Pages 1–9 for a complete description). The structure consists of a III-V mesa coated with a SiN layer and a narrow Si waveguide with the QCL region according to the structure described in [73]. Figure 1e shows the cross-section of the reported DFB-QCL laser on Si [65], emitting quasi-TM light around 7.4 μm, with its optical mode as shown in Figure 1f. The structure consists of III-V materials InP on Si, with a QCL region made from a GaInAs/AlInAs heterostructure designed according to [74,75]. The simulated optical modes of the three laser sources clearly affirm that the optical mode size and polarizations can be significantly different depending on the lasing structure designs.

### 2.2. As_2_Se_3_-on-CaF_2_ Waveguides

**Figure 2:**
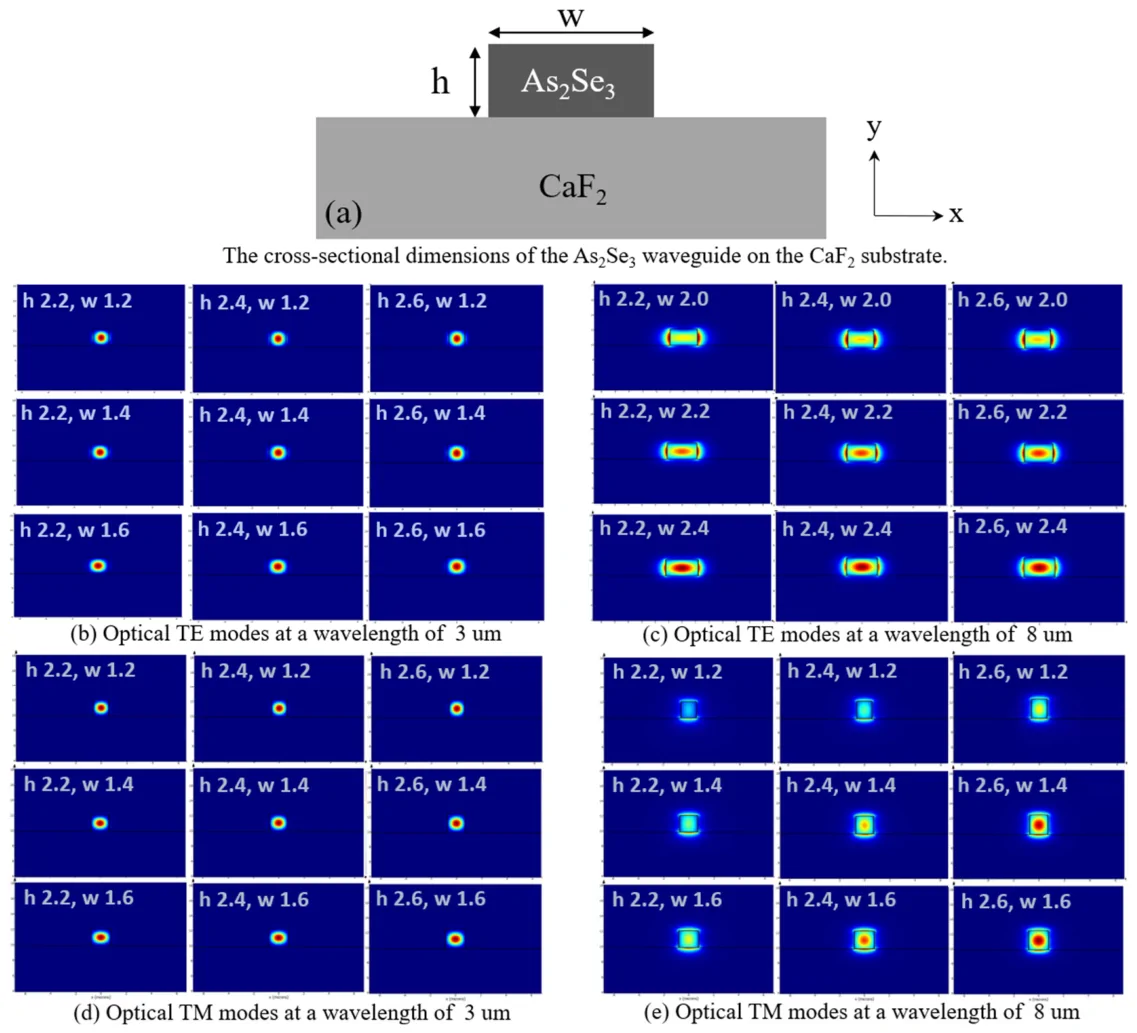
(**a**) Cross-section of an As_2_Se_3_-on-CaF_2_ waveguide. Investigation of the waveguide optical modes for different values of waveguide thickness (h) and width (w) for the shortest and longest wavelengths of interest at (**b**,**d**) 3 µm and (**c**,**e**) 8 µm in both TE and TM polarizations.

To initially investigate the potential of using an As_2_Se_3_-on-CaF_2_ waveguide platform to accommodate direct integration with the three different laser sources across the mid-IR 3–8 µm spectral range, the ability of the chalcogenide waveguide to support proper optical mode confinement needs to be carefully examined over such a relatively wide spectral range. Figure 2a shows a typical cross-section of an As_2_Se_3_-on-CaF_2_ waveguide; the optical confinement can be achieved using As_2_Se_3_ [76] owing to its higher refractive index relative to CaF_2_ [77] over the entire wavelength of interest (see Supplementary File Pages 10–11) and the low-loss characteristics of both materials, which is consistent with previously reported related work [41]. Both As_2_Se_3_ (transparent from ~0.8 µm to beyond 15 µm) and CaF_2_ (transparent up to ~8–9 µm) are transparent across the entire 3–8 µm range [38,78,79,80]. Conventional platforms cannot cover this range because the SiO_2_ undercladding of silicon-on-insulator absorbs strongly beyond ~4 µm, and silicon or germanium waveguides on silicon substrates suffer absorption at longer wavelengths [3]. In addition, the high index contrast between As_2_Se_3_ (*n* ≈ 2.8) [78] and CaF_2_ (*n* ≈ 1.35–1.42) [77] allows the mode size, effective index, and polarization response to be tuned over a wide range by changing only the waveguide width. It is important to mention that As_2_Se_3_ has a glass transition temperature of about 180 °C [78,81], well above the typical operating temperatures of gas sensors for environmental, industrial, and agricultural emission monitoring [1]. The CaF_2_ substrate is thermally stable, and its thermal conductivity [79] is almost 50 times higher than that of As_2_Se_3_ [81], so it can act as an efficient heat sink for the waveguide core. Optical heating is expected to be limited because the photon energies at 3–8 µm are far below the As_2_Se_3_ bandgap (~1.7–1.8 eV) [78,80], which makes band-edge and two-photon absorption negligible, and because QCL- and ICL-based gas sensing typically requires only a few milliwatts of optical power [69,82]. It is also important to mention that practical realization of As_2_Se_3_-on-CaF_2_ waveguides involves several fabrication challenges, which could be addressed with established chalcogenide processing techniques. As_2_Se_3_ films can be deposited by thermal evaporation, sputtering, or pulsed laser deposition, followed by annealing steps to stabilize their stoichiometry and refractive index [78,80]. The close thermal expansion match between As_2_Se_3_ [83] and CaF_2_ [79] helps reduce stress-induced delamination at the interface. In addition, careful development of the waveguide patterning process is needed to keep sidewall roughness low [84,85]. Regarding a possible route toward integration on silicon, besides wafer bonding, As_2_Se_3_ could also be deposited at low temperatures, making it potentially compatible with back-end-of-line processing, provided that the process involving arsenic could be managed via dedicated tools [38].

To verify the ability of the As_2_Se_3_-on-CaF_2_ waveguide platform to accommodate all relevant optical wavelengths at the same As_2_Se_3_ waveguide thickness, Figure 2b–e shows an investigation of the waveguide optical modes for different values of waveguide thickness (h) and width (w), at which the structures can maintain optical mode confinement for the shortest (3 µm) and longest (8 µm) wavelengths of interest in both polarizations. As shown in Figure 2e, at the longest wavelength of 8 µm, the thinnest waveguide thickness that can support proper optical mode confinement of the quasi-TM light is h ~ 2.4 µm with a waveguide width (w) of 1.6 µm. For quasi-TE light at 8 µm wavelength, as shown in Figure 2c, the same waveguide thickness (h) of 2.4 µm can be interestingly used to allow optical mode confinement with a slightly wider waveguide width (w) of 2.4 µm. In addition, for the shorter wavelength of 3 µm, a 2.4 µm thick waveguide can also be used to support optical mode confinement at a consistent waveguide width of around 1.6 µm. Therefore, from the study described in this part, we found that employing an As_2_Se_3_-on-CaF_2_ waveguide could excellently accommodate proper optical mode confinement across optical wavelength ranges from 3 to 8 µm while maintaining the same As_2_Se_3_ waveguide thickness.

## 3. Investigation of Butt-Coupling Integration Between Three Different Mid-IR Laser Sources on Si and As_2_Se_3_-on-CaF_2_ Passive Waveguide Platform

To demonstrate the use of an As_2_Se_3_-on-CaF_2_ waveguide platform over a wide mid-IR wavelength range from 3 to 8 µm, the integration of the three different mid-IR laser sources on Si with the As_2_Se_3_-on-CaF_2_ chalcogenide waveguide was investigated across the optical wavelengths. The potential to obtain a good optical coupling efficiency and strong sensing figure-of-merit performance, together with acceptable alignment tolerance of the integration scheme, was thoroughly investigated.

### 3.1. Coupling Efficiency

**Figure 3:**
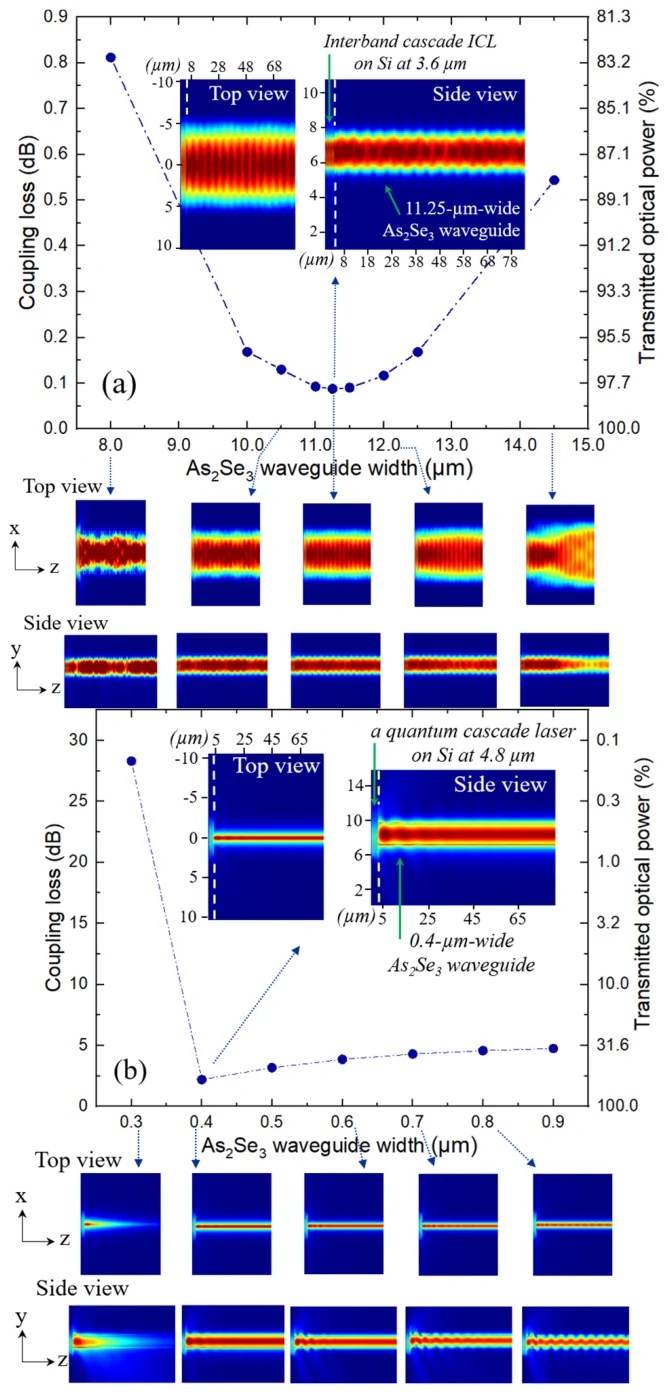

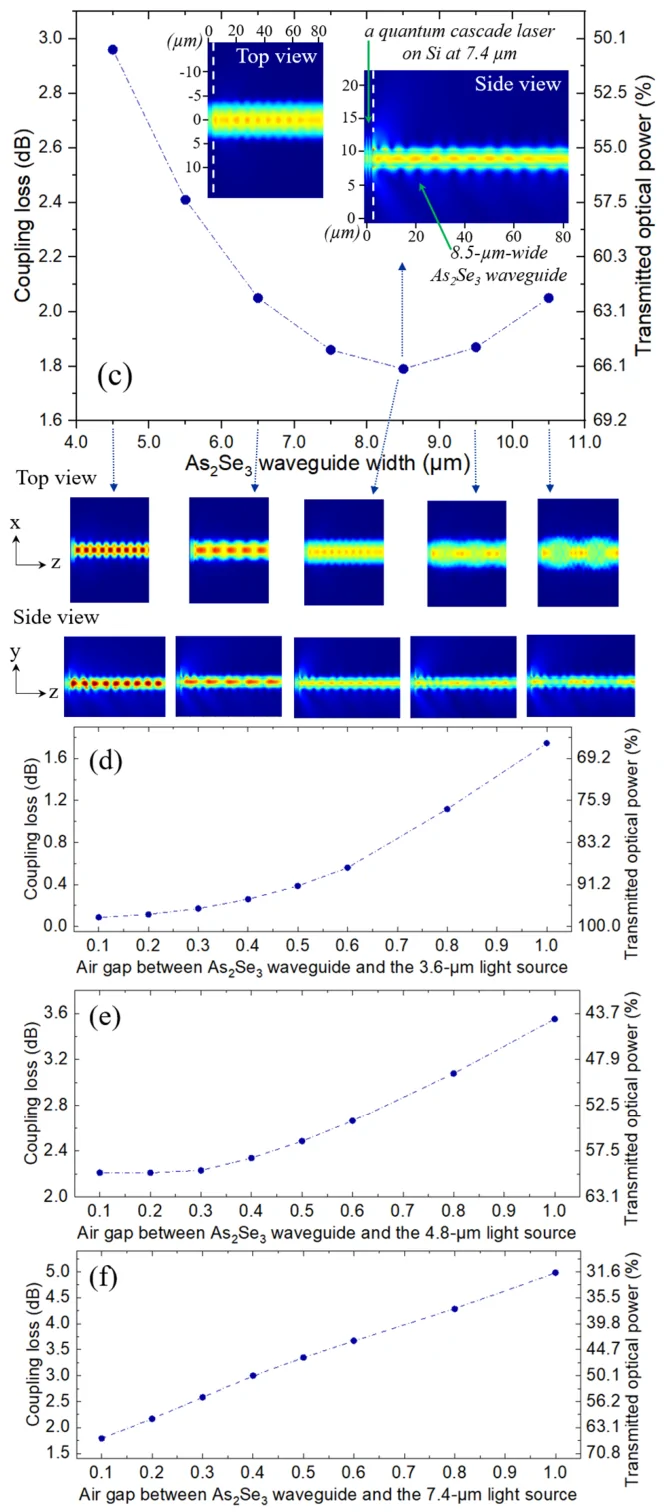
(**a**) Coupling efficiency between the laser optical mode and the fundamental guided mode in an As_2_Se_3_-on-CaF_2_ waveguide at different waveguide width values with (**a**) the interband cascade ICL on Si [63], (**b**) the quantum cascade laser (QCL) on Si [64], and (**c**) the DFB-QCL laser on Si [65]. (**d**–**f**) Coupling efficiency with the air gap between the light sources and the waveguide structure increasing from 0.1 to 1 µm. **Top view**: Top-down view of the device along the propagation direction (x–z plane). **Side view**: Longitudinal cross section representing a slice along the propagation direction (y–z plane).

To verify whether the 2.4 µm thick As_2_Se_3_-on-CaF_2_ waveguide platform chosen based on the previous section results could be effectively employed to accommodate direct optical coupling with the three different mid-IR laser sources, this part of the study investigated the direct coupling efficiency between the two of them. Although a close end-fire coupling (no air gap) would provide the best optical coupling condition [86] and is practically possible [87], in all the cases reported in this work, we assume a 100 nm (0.1 µm) air gap between the light source and the waveguide structure to be conservative. This can be considered consistent with the achievable condition of integration between a semiconductor device and amorphous waveguide material that could be flexibly prepared using the evaporation technique [87,88], such as As_2_Se_3_ and CaF_2_ [89,90,91]. For the first case of the ICL on Si [63], emitting quasi-TE light around 3.6 μm, Figure 3a shows that a 2.4 µm thick and 11.25 µm wide As_2_Se_3_-on-CaF_2_ waveguide can enable a low-loss optical butt-coupling of as low as ~0.09 dB (> 97% optical transmission) between the laser optical mode (Figure 1b) and the fundamental guided mode in the waveguide. As the waveguide width deviated from the optimized value of 11.25 µm, the coupling efficiency only gradually decreased because the waveguide width was relatively large, and it took a ±3.25 µm width variation to cause an approximately 0.4–0.8 dB reduction in the optical transmission. Due to the required large waveguide width, which is much larger than the condition discussed in Figure 2, optical mode beating between the fundamental optical mode and the other unintentionally excited horizontal higher-order modes in the waveguide is clearly observed from the top-view optical propagation profiles (which will be later addressed by tapering in Section 3.2). In particular, the beating is more evident when the waveguide is excessively wide (w = 14.5 µm) or overly narrow (w = 8 µm), indicating that a large deviation in the waveguide width from the optimal value actually leads to stronger excitation of horizontal higher-order optical modes in the waveguide, responsible for the decreased coupling efficiency.

For the second case of the quantum cascade laser (QCL) on Si [64], emitting quasi-TM light around 4.8 μm, Figure 3b shows that a 2.4 µm thick and 0.4 µm wide As_2_Se_3_-on-CaF_2_ waveguide can enable a low-loss optical butt-coupling of as low as ~2.2 dB (60% optical transmission) between the laser optical mode (Figure 1d) and the fundamental guided mode in the waveguide. Contrary to the first case, a much narrower waveguide width of 0.4 µm is found to be required for optimized coupling efficiency because the optical mode from the second laser (Figure 1d) is much more confined than that of the first laser (Figure 1b). Nevertheless, the more confined optical mode of the laser design generally leads to a higher optical coupling loss between the two structures, as we also found in previous work [86]. Nevertheless, decreasing the waveguide width to 0.3 µm appears to abruptly increase the optical coupling loss to 28 dB (only 0.15% optical transmission) because the 0.3 µm-wide waveguide cannot support the optical mode anymore, as shown in the top- and side-view propagation profile inset. On the other hand, increasing the waveguide width also gradually increases the optical coupling loss; the unintentionally excited vertical higher-order modes in the waveguide are increasingly observable from the side-view optical propagation profiles as the waveguide width increases to more than 0.6 µm.

For the third case of a DFB-QCL laser on Si [65], emitting quasi-TM light around 7.4 μm, Figure 3c shows that a 2.4 µm thick and 8.5 µm wide As_2_Se_3_-on-CaF_2_ waveguide can enable a low-loss optical butt-coupling of as low as ~1.8 dB (66% optical transmission) between the laser optical mode (Figure 1e) and the fundamental guided mode in the waveguide. For compatibility with the larger optical mode size of the laser design (Figure 1f) compared to the second case (Figure 1d), a relatively large waveguide width of 8.5 µm is found to be suitable for optimized coupling efficiency. Moreover, consistent with the previous two cases, using a waveguide that is either too narrow or too wide will decrease the coupling efficiency. Accordingly, as in the inset, the optical mode beating between the fundamental optical mode and the other unintentionally excited horizontal higher-order modes in the waveguide becomes more pronounced as the waveguide width deviates from the most suitable values, affirming that the optical mode size mismatch between the laser source and the waveguide is very influential in determining the optical coupling efficiency and in minimizing the unintentional excitation of the higher-order modes in the waveguide. In addition, Figure 3d–f shows the coupling efficiency with the air gap between the light sources and the waveguide structure increasing from 0.1 µm up to 1 µm. To maintain the coupling loss lower than 3 dB for all three light sources, a gap of 0.3 µm should be used. Although this could be promisingly within the limit of the theoretical interface gap based on a recent study using the flip-chip technique [59], optical integration between a semiconductor device and amorphous waveguide material that could be flexibly prepared using the evaporation technique such as As_2_Se_3_ and CaF_2_ [89,90,91] can be achieved with a gap of less than 100 nm [57,88]. It is important to note that in practice, coupling performance will further depend on facet preparation and dicing quality, angular misalignment, bonding and packaging accuracy. In addition, reflection may cause optical feedback into the laser, which may require antireflection coatings on the laser facet. Addressing these challenges will be essential for practical chip-scale integration and is the subject of future work.

From this part of the study, we promisingly found that an As_2_Se_3_-on-CaF_2_ waveguide platform with the same waveguide thickness of 2.4 µm can be employed to accommodate optical coupling with the widely different lasing optical mode sizes and polarizations from various mid-IR laser sources depending on the laser designs, by carefully adjusting only the waveguide width. We would also like to note that when the waveguide is wide enough to support more than one mode, the coupling loss shown on the left vertical axis of Figure 3a,c arises mainly from light coupled into higher-order modes. The results indicate that the mode-size mismatch between the laser output and the waveguide strongly affects both coupling efficiency and higher-order mode excitation. As the waveguide width deviates from its optimal value, whether wider or narrower, higher-order modes are increasingly excited. In the direct coupling scheme, therefore, choosing the optimal waveguide width is crucial, as it maximizes mode matching between the laser output and the waveguide’s fundamental mode while minimizing power lost to higher-order modes.

### 3.2. Linear Waveguide Taper Design to Enable Competitive Sensing Figure-of-Merit

As described in the previous section, although we have found that an As_2_Se_3_-on-CaF_2_ waveguide platform with a homogeneous thickness of 2.4 µm can be used to enable direct end-fire optical coupling with different mid-IR laser sources across the 3–8 µm spectral range, with varied lasing optical mode sizes and polarizations, the required waveguide widths are relatively large for the case of mid-IR laser sources on Si emitting at the 3.6 and 7.4 µm optical wavelengths. Such a wide waveguide could lead not only to the possible excitation of a higher-order mode as discussed in the previous section, but also to a lower gas detection performance because most of the optical mode is found to be confined in the waveguide material instead of in the surrounding air. In particular, for waveguide-based spectroscopic absorption-traced gas sensing, the value of Γ = ∂*n_mode_*/∂*n_surrounding_*, which evaluates the sensitivity of the waveguide with respect to the change in the surrounding refractive index (*n_surrounding_*), has become the preferred figure of merit (FOM) employed to compare different waveguide-based sensing structures [3,35,56,66], while *n_mode_* represents the effective index of the guided optical mode. To resolve the mentioned problems, in this part of the study, the design of an As_2_Se_3_-on-CaF_2_ waveguide with a taper section was evaluated in order to verify the possibility of optical waveguide sensing with a competitive value of Γ.

**Figure 4:**
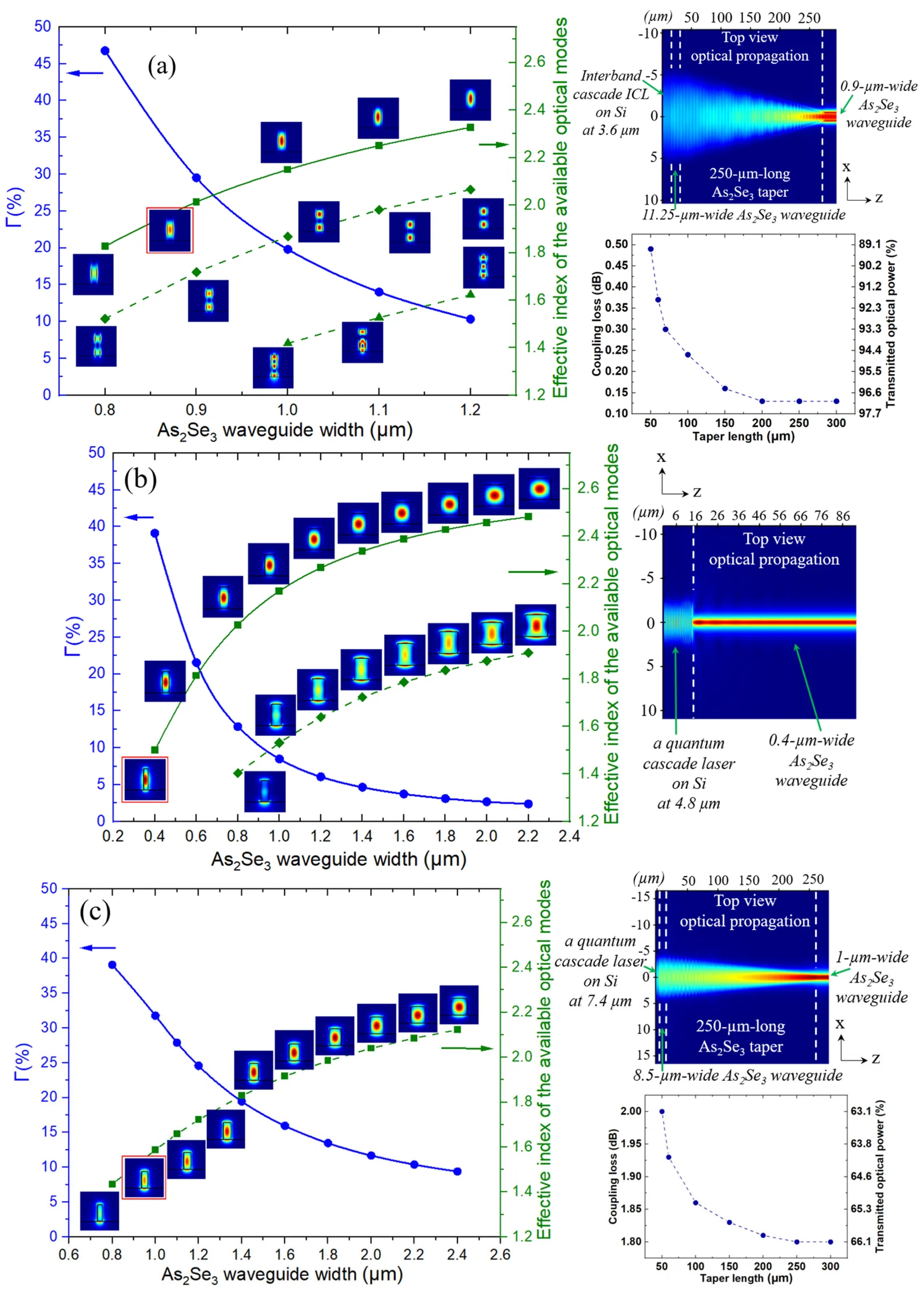
The Γ values of the As_2_Se_3_-on-CaF_2_ waveguide at different As_2_Se_3_ waveguide widths (blue lines) and the supported waveguide optical modes (green lines) for (**a**) a quasi-TE 3.6 µm optical wavelength obtained from the ICL on Si [63], (**b**) a quasi-TM 4.8 μm optical wavelength from the quantum cascade laser (QCL) on Si [64], and (**c**) a quasi-TM 7.4 μm optical wavelength from the DFB-QCL laser on Si [65]. The corresponding inset shows efficient optical propagation (**a**) from the external laser chip at 3.6 µm to the 11.25 µm wide As_2_Se_3_ waveguide via a 250 µm long taper section to the 0.9 µm wide As_2_Se_3_ waveguide, (**b**) from the external laser chip at 4.8 µm directly to the 0.4 µm wide As_2_Se_3_ waveguide without a taper, and (**c**) from the external laser chip at 7.4 µm to the 8.5 µm wide As_2_Se_3_ waveguide via a 250 µm long taper section to the 1 µm wide As_2_Se_3_ waveguide. It is important to mention that the reported transmitted optical power in the inset of (**a**,**c**) concerns only the desired fundamental sensing mode at the As_2_Se_3_ waveguide.

For the first case of the ICL on Si [63] emitting quasi-TE light around a 3.6 µm optical wavelength, as in Figure 4a (blue line), the Γ values of the As_2_Se_3_-on-CaF_2_ waveguide can be significantly improved to more than 29% as the width of the As_2_Se_3_ waveguide is reduced to 0.9 µm. This is consistent with the fact that a lower waveguide dimension leads to a less optically confined waveguide, and hence a higher optical overlap with the surrounding air. As shown in the right inset with field profiles and taper-length convergence provided in the lower part, we found that a 250 µm-long taper section of the As_2_Se_3_ waveguide can be used to efficiently transfer the desired optical mode from the 11.25 µm-wide As_2_Se_3_ waveguide required for optimized coupling efficiency with the external laser chip to the 0.9 µm-wide As_2_Se_3_ waveguide required for competitive values of Γ for spectroscopic absorption sensing applications. Significantly, the tapering structure allows us to maintain the coupling efficiency of approximately 97% optical transmission to the 11.25 µm-wide waveguide (Figure 3a) at the much narrower waveguide of 0.9 µm shown in the Figure 4a right inset. It should be noted that, although a 0.9 µm-wide As_2_Se_3_ waveguide can support more than one optical mode as in Figure 4a (green line), only the fundamental mode is excited with proper alignment between the external laser and the waveguide platform, as in the right inset, of which the effect of horizontal and vertical misalignment will be further elucidated in the next section.

For the second case of the quantum cascade laser (QCL) on Si [64] emitting quasi-TM light around 4.8 μm, the 0.4 µm-wide As_2_Se_3_ waveguide required for optical coupling with the external laser chip (Figure 3b) can already render competitive Γ values of 39% as in Figure 4b (blue line). Moreover, owing to its compactness, the 0.4 µm-wide waveguide can support only the fundamental quasi-TM optical mode (green line). Therefore, a direct end-fire optical coupling between the external laser chip and a 0.4 µm-wide As_2_Se_3_ waveguide can be conveniently employed as in the right inset of Figure 4b without the requirement for an additional tapering structure.

For the third case of a DFB-QCL laser on Si [65] emitting quasi-TM light around 7.4 μm, as in Figure 4c (blue line), the width of the As_2_Se_3_ waveguide needs to be reduced to 1 µm to attain a competitive Γ value of approximately 32%. Therefore, as in the right inset with field profiles and taper-length convergence provided in the lower part, a 250 µm long taper section of the As_2_Se_3_ waveguide is required to transfer the optical mode from the 8.5 µm wide As_2_Se_3_ waveguide needed for optimized coupling efficiency (Figure 3c) with the external laser chip to the 1 µm wide As_2_Se_3_ waveguide for competitive values of Γ. It should be mentioned that, at this relatively large wavelength, the waveguide can naturally support only the fundamental mode, as shown in Figure 4c (green line). Owing to the tapering structure, the coupling efficiency of approximately 66% optical transmission to the 8.5 µm wide waveguide (Figure 3c) can be maintained at the much narrower waveguide of 1 µm shown in Figure 4c’s right inset.

From the study in this part, we promisingly find that an As_2_Se_3_-on-CaF_2_ waveguide platform with the same waveguide thickness of 2.4 µm can be employed to enable competitive values of Γ for spectroscopic absorption sensing applications across optical wavelength ranges from 3 to 8 µm. In particular, when relatively wide As_2_Se_3_ waveguides are required for optimized coupling efficiency with the external laser chip, as in the cases of an ICL on Si [63] at 3.6 µm and a DFB-QCL laser on Si [65] at 7.4 μm, a tapering structure of the waveguide platform can be employed to allow efficient optical transmission from the wide As_2_Se_3_ waveguide to the much narrower waveguide required for spectroscopic sensing with a competitive sensing figure of merit, Γ. Additionally, we affirm that, at the taper output in Figure 4a,c, essentially all of the guided power is in the fundamental mode, with negligible power in higher-order modes. For all three cases, the 2.4 µm thick As_2_Se_3_-on-CaF_2_ waveguide platform allows for impressive Γ values of ~30% across the spectral range of interest, which are comparable to the recently optimized state-of-the-art devices [35,36,66].

Last but not least, it is important to assess the optical loss due to potential sidewall roughness in waveguides used for sensing applications [66,92]. To this end, we employed 3D-FDTD simulations to evaluate the scattering loss in the waveguide. Following the method proposed by [93], the optical mode was launched at each corresponding wavelength, and two power monitors were placed along the propagation path: one at the input to record the total transmitted power, and a second after a 100 µm propagation length, allowing the loss to be deduced in dB per unit length [93]. Given that thermally evaporated and dry-etched As_2_Se_3_ chalcogenide waveguides can exhibit RMS sidewall roughness as low as 1.5 nm [85], we adopted this value in our simulations. The 2.4 µm thick As_2_Se_3_ waveguide—designed to maximize the sensing figure of merit at each wavelength considered—supports optical propagation with a loss of approximately 2 dB/cm for quasi-TE light, and below 1 dB/cm for quasi-TM light (see Supplementary File Page 12). These results are consistent with loss values reported in other chalcogenide waveguide studies [94].

### 3.3. Alignment Tolerance of the Integration Scheme

**Figure 5:**
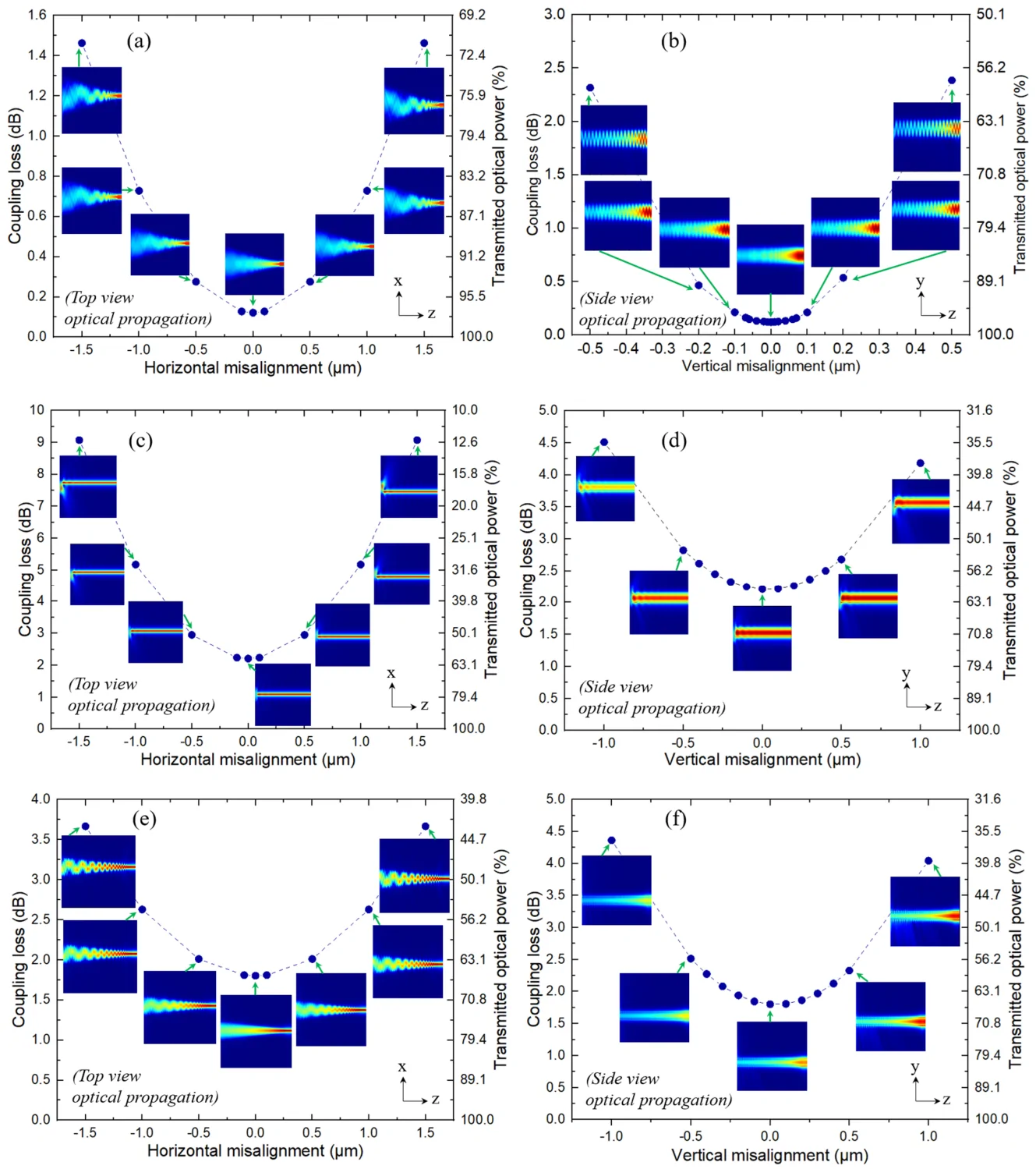
The effect of the horizontal and vertical positional fabrication misalignment between the 2.4 µm thick As_2_Se_3_-on-CaF_2_ waveguide proposed in the previous section and (**a**,**b**) the ICL on Si [63] at 3.6 µm, (**c**,**d**) the quantum cascade laser (QCL) on Si [64] at 4.8 µm, and (**e**,**f**) the DFB-QCL laser on Si [65].

Figure 5 reports the effects of the vertical and horizontal positional fabrication misalignment between the external laser chip on Si and the As_2_Se_3_-on-CaF_2_ waveguide platform for the three different cases across the optical wavelength of interest. In all cases, vertical and horizontal positional misalignment of ±100 nm (0.1 µm) can be tolerated by the investigated end-fire optical coupling scheme. The horizontal misalignment will cause the higher-order mode to be excited at the 11.25 µm-wide (Figure 5a) and 8.5 µm-wide (Figure 5e) As_2_Se_3_ waveguides for the optical coupling with the ICL on Si [63] at 3.6 µm and a DFB-QCL laser on Si [65] at 7.4 μm, respectively, as observed from the top-view optical propagation. For the case of the direct optical coupling between the quantum cascade laser (QCL) on Si [64] at 4.8 µm and a 0.4 µm-wide As_2_Se_3_ waveguide (Figure 5c), the horizontal misalignment will rapidly decrease the optical overlap between the laser and the waveguide once the misalignment is higher than 0.5 µm. For the vertical misalignment, at the shorter wavelength of 3.6 µm, it will cause the higher-order modes to be excited once the misalignment approaches 0.2 µm (Figure 5b). On the contrary, for the longer wavelengths of 4.8 and 7.4 µm, the vertical misalignment will not lead to higher-order mode excitations (Figure 5d,f), which is consistent with Figure 4b,c, showing that the 2.4 µm thick As_2_Se_3_ waveguides can support only one vertical mode at these wavelengths. For both cases, the vertical misalignment will rapidly decrease the optical coupling efficiency once the misalignment is higher than 0.5 µm. It should be mentioned that the end-fire optical coupling misalignment tolerance of ±0.1–0.5 µm in the mid-IR region (3–8 µm) is more relaxed compared to 25–50 nm in the near-IR region found in our previous studies [56,86], consistent with a larger wavelength dimension.

## 4. Conclusions and Remarks

This study has shown that an As_2_Se_3_-on-CaF_2_ waveguide platform can potentially accommodate optical integration with three different mid-IR light sources on Si emitting at wavelengths of 3.6, 4.8, and 7.4 μm while maintaining competitive waveguide-based sensing performance. Despite a significant difference in lasing optical mode size and polarization among different laser structure designs, we found that the As_2_Se_3_-on-CaF_2_ waveguide can be employed to accommodate efficient butt-coupling with all of them, while the same As_2_Se_3_ waveguide thickness can be maintained, allowing the same As_2_Se_3_-on-CaF_2_ waveguide platform with a carefully varied width to be employed to achieve both good optical coupling performance and competitive sensing figure of merit over the entire wavelength range of interest, using top–down optical lithography and dry etching techniques. Moreover, as discussed in Section 2.2, a waveguide thickness of 2.4 µm was chosen because it is the minimum thickness that provides adequate optical mode confinement for quasi-TM light at the longest wavelength of 8 µm. Specifically, we found the following: (1) The optical mode size mismatch between the laser source and the waveguide strongly influences the coupling efficiency and higher-order mode excitation; as shown in Figure 3, changing the waveguide width from its optimal value—whether too wide or too narrow—increasingly excites higher-order modes. Simulated results of various example designs demonstrate that, to integrate an ICL on Si [63] at 3.6 µm, a quantum cascade laser (QCL) on Si [64] at 4.8 µm, and a DFB-QCL laser on Si [65] at 7.4 μm, the optimal As_2_Se_3_ waveguide width for high coupling efficiency should be 11.25, 0.4, and 8.5 μm, respectively, while the waveguide thickness for all cases is maintained at 2.4 μm. Notably, a much narrower waveguide width of 0.4 µm is required for optimized coupling efficiency with the second laser because of the more confined optical mode of this particular laser design. (2) When relatively wide As_2_Se_3_ waveguides are required for optimized coupling efficiency with the external laser chip as in the cases of an ICL on Si [63] at 3.6 µm and a DFB-QCL laser on Si [65] at 7.4 μm, a tapering structure of the waveguide platform can be employed to allow efficient optical transmission from the wide As_2_Se_3_ waveguide to the much narrower (0.9–1 μm) waveguide required for spectroscopic sensing with competitive sensing figure of merit, Γ, as shown in Figure 4. Notably, across all three cases, the 2.4 µm thick As_2_Se_3_-on-CaF_2_ waveguide platform achieves Γ values of ~30%, on par with recently reported state-of-the-art devices [35,36]. (3) As shown in Figure 5, in all cases, the end-fire coupling scheme exhibits a much larger misalignment tolerance (±100–500 nm) in the mid-IR range compared to the ±25–50 nm reported for near-IR devices in our previous work [56,86], reflecting the benefit of larger mode dimensions at longer wavelengths. Table 1 presents a comparative summary of various inter-chip optical integration works reported in the literature alongside this work. Whereas previous related works have focused on optical integration over a narrow wavelength range for telecommunications or optical interconnects [53,54,55,88], or for spectroscopic sensing [56,57,58,59,62], to be potentially compatible with spectroscopic techniques such as fingerprint absorption sensing, this work has firstly investigated and proposed a chalcogenide As_2_Se_3_-on-CaF_2_ waveguide platform for wideband operation across the important mid-IR wavelength range of 3–8 µm. The studies have demonstrated the attractiveness of the chalcogenide As_2_Se_3_-on-CaF_2_ waveguide platform in terms of design flexibility to obtain both high coupling efficiency and a high spectroscopic sensing figure of merit, concerning integration with different mid-IR light sources on Si with significantly different optical mode sizes and polarizations.

## Figures and Tables

**Table 1:** Comparative table between this work and prior research on inter-chip optical integration.

Study	Materials and Waveguide Platform	Wavelength Range and Potential Application	Coupling Efficiency (Coupling Loss)	Key Distinction vs. This Work
This work	Three different mid-infrared (mid-IR) light sources at λ of 3.6, 4.8, and 7.4 μm, and As_2_Se_3_-on-CaF_2_ waveguide, fixed 2.4 µm thickness	3–8 µm (Spectroscopic sensing: mid-infrared)	~0.1–2 dB	
Ref. [53] (Theurer et al., 2020)	InP laser chip, and Si_3_N_4_-on-SiO_2_ waveguide	1.530–1565 µm (Telecommunication)	~2.1 dB	Near-infrared wavelength: chip-scale optical interconnect application
Ref. [54] (Boust et al., 2020)	InGaAsP/InP laser chip, and Si_3_N_4_-on-SiO_2_ waveguide	~1.576 µm (Telecommunication)	NA	Near-infrared wavelength: comb generation application
Ref. [55] (Lihachev et al., 2022)	InP laser chip, and Si_3_N_4_-on-SiO_2_ waveguide	~1.55 µm (LiDAR)	NA	Near-infrared wavelength: coherent ranging application
Ref. [56] (Jaturaphagorn et al., 2021)	GaSb-based superluminescent on chip, and SiN-on-SiO_2_ waveguide	~2 µm (Spectroscopic sensing: short-wave infrared)	~1.25 dB	Single wavelength in short-wave infrared
Ref. [57] (Paparella et al., 2022)	GaSb laser diode on chip, and polymer-based waveguide	~2.3 µm (Spectroscopic sensing: short-wave infrared)	~2–6 dB	Narrow wavelength range in short-wave infrared
Ref. [58] (Koompai, 2024)	InAs/AlSb QCL, and Ge-rich SiGe waveguide on graded buffer	~7.4 µm (Spectroscopic sensing: mid-infrared)	~4 dB	Single wavelength in mid-infrared
Ref. [59] (Wang et al., 2024)	InGaAs/AlInAs QCL, and Ge-on-Si waveguide	~7.2 µm (Spectroscopic sensing: mid-infrared)	~2 dB	Single wavelength in mid-infrared
Ref. [62] (Radosavljevic et al., 2018)	III-V Gain chip, and Ge-on-SOI waveguide	5.2–5.3 µm (Spectroscopic sensing: mid-infrared)	~4 dB	Single wavelength: filter application
Ref. [88] (Chatzitheocharis et al., 2020)	InP-Quantum-Dot (QD) laser/SiN_4_-on-SiO_2_ waveguide	1.55 µm (Telecommunication)	~0.6 dB	Near-infrared wavelength: Interposer strategy between chips

## Data Availability

The data presented in this study are available upon request from the corresponding author.

## Supplementary Materials

The following supporting information can be downloaded at: https://www.mdpi.com/article/10.3390/s26206632/s1.
