# Supplementary material for: FDTD Analysis of End-Fire Coupling Between Mid-IR On-Chip Light Sources on Si and As2Se3-on-CaF2 Waveguide from 3 to 8 µm for Spectroscopic Sensing
Source: Sensors (Basel). 2026 Oct 20;26(20):6632. doi: 10.3390/s26206632 (PMC13708912; doi:10.3390/s26206632)
Supplement: Supplementary file 1 [file sensors-26-06632-s001.zip › sensors-4578554-supplementary.pdf]

Employed material parameters for reported interband cascade ICL structure on Si [63] at 3.6 um

Table 2. III-V Layers

Layers	Material	Thickness (nm)	Doping (cm^{-3})
Substrate	GaSb	–	–
Buffer	*n*-GaSb	400	1.0×10^{17}
Etch stop	*n*-InAsSb	1000	5.0×10^{18}
Sacrificial	*n*-GaSb	100	1.0×10^{17}
Top contact	*n*-InAsSb	100	5.0×10^{18}
Transition	*n*-InAs/AlInSb SL	33.8	5.0×10^{18}
Transition	*n*-InAs/AlSb SL	31.9	5.0×10^{18}
Top clad	*n*-InAs/AlSb SL	1400	$1.5–5.0 \times 10^{17}$
Transition	*n*-InAs/AlGaSb SL	24.5	2.0×10^{17}
Top SCH	*n*-GaSb	600	$0.05–1.0 \times 10^{17}$
Transition	*n*-InAs/AlSb SL	20	2.0×10^{18}
Active	InAs/AlSb/GaInSb	290	Various
Transition	*n*-InAs/AlSb SL	20	2.0×10^{17}
Bottom SCH	*n*-GaSb	600	$0.05–1.0 \times 10^{17}$
Transition	*n*-InAs/AlSb SL	24.5	2.0×10^{17}
Bottom contact	*n*-InAs/AlSb SL	200	5.0×10^{17}
Bonding SL	GaSb/AlSb SL	30	–
Capping layer	GaSb	10	–

Material	Refractive index (real)	Ref.
InAs	3.5498+i0.015623	Adachi 1989 (ref)
AlSb	3.1659+i0.020259	Adachi 1990 (ref)
InSb	3.8993+i0.10288	Adachi 1989 (ref)
GaSb	3.7235+i0.019379	Adachi 1989 (ref)
$Ga_{0.65}In_{0.35}Sb$	3.78503+i0.04860	0.65*GaSb+0.35*InSb
InAsSb	3.72455+i0.05925	0.5*InAs+0.5*InSb
AlInSb	3.5326+i0.06157	0.5*AlSb+0.5*InSb
AlGaSb	3.4447+i0.019819	0.5*AlSb+0.5*GaSb
GaInSb	3.8114+i0.06113	0.5*GaSb+0.5*InSb
ICL	3.478624+i0.012359	Link for the data
n-InAs/AlInSb	3.5412+i0.0386	0.5*InAs+0.5*AlInSb
n-InAs/AlSb	3.3578+i0.0179	0.5*InAs+0.5*AlSb
n-InAs/AlGaSb	3.4972+i0.0177	0.5*InAs+0.5*AlGaSb

Employed material parameters for reported interband cascade ICL structure on Si [63] at 3.6 um

Details on the treatment of the active laser regions

ICL structure (1 stage) In this paper, 7-stage was applied

The layer structure of one period starting with the barrier separating the electron injector and the active region is as follows: 25 Å AlSb/17 Å InAs/30 Å $Ga_{0.65}In_{0.35}Sb$/14 Å InAs/10 Å AlSb/30 Å GaSb/10 Å AlSb/45 Å GaSb/25 Å AlSb/42 Å InAs/12 Å AlSb/32 Å InAs/12 Å AlSb/25 Å InAs/12 Å AlSb/20 Å InAs/12 Å AlSb/17 Å InAs/12 Å AlSb/17 Å InAs

1 period of ICL layer around 419 A (41.9 nm) or (=293.3 nm for 7 stages)
Refractive index for 1 period.

d, thickness (nm)	n, real part	k, imaginary part	n*d	k*d
2.5	3.1659	0.020259	7.91475	0.050648
1.7	3.5498	0.015623	6.03466	0.026559
3	3.78503	0.0486	11.35509	0.1458
1.4	3.5498	0.015623	4.96972	0.021872
1	3.1659	0.020259	3.1659	0.020259
3	3.7235	0.019379	11.1705	0.058137
1	3.1659	0.020259	3.1659	0.020259
4.5	3.7235	0.019379	16.75575	0.087206
2.5	3.1659	0.020259	7.91475	0.050648
4.2	3.5498	0.015623	14.90916	0.065617
1.2	3.1659	0.020259	3.79908	0.024311
3.2	3.5498	0.015623	11.35936	0.049994
1.2	3.1659	0.020259	3.79908	0.024311
2.5	3.5498	0.015623	8.8745	0.039058
1.2	3.1659	0.020259	3.79908	0.024311
2	3.5498	0.015623	7.0996	0.031246
1.2	3.1659	0.020259	3.79908	0.024311
1.7	3.5498	0.015623	6.03466	0.026559
1.2	3.1659	0.020259	3.79908	0.024311
1.7	3.5498	0.015623	6.03466	0.026559
Average refractive index			3.478624	0.012359

Simulation structures and monitor positions used for verification along the optical part for the integration between interband cascade ICL structure on Si [63] and at 3.6 um

(from the external laser chip at 3.6 μm to the 11.25 μm wide As_2Se_3 waveguide via a 250 μm long taper section to the 0.9 μm wide As_2Se_3 waveguide)

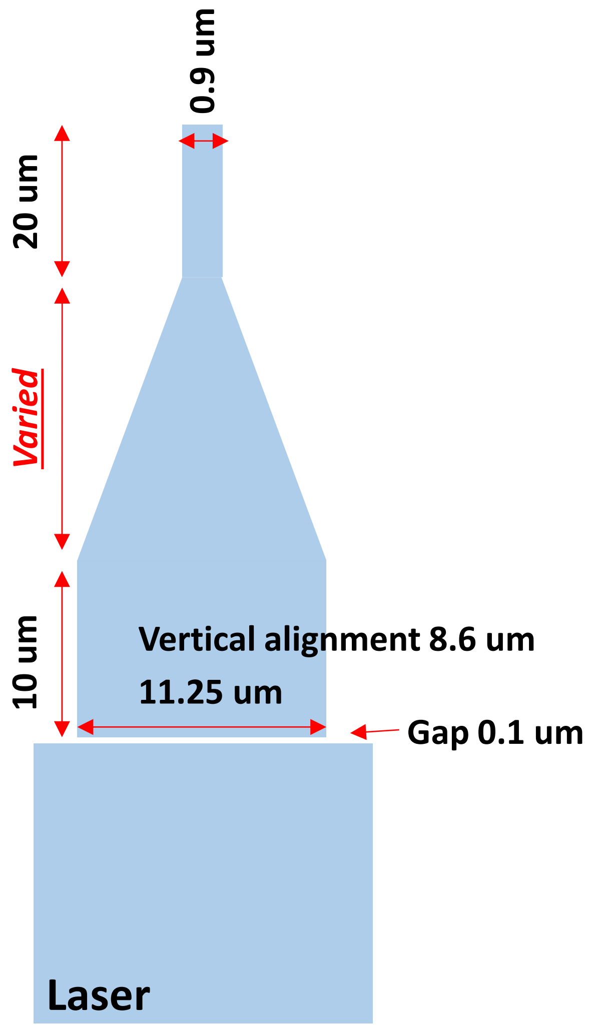

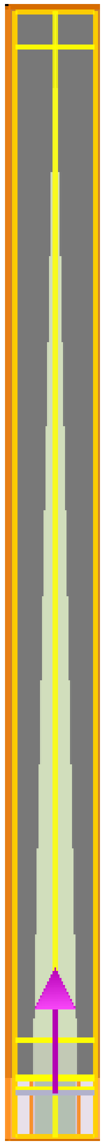

Power monitor at specified mode at the 0.9 μm wide As_2Se_3 waveguide for the reported transmitted optical power in the desired fundamental sensing mode at the As_2Se_3 waveguide with respect to the power from the input.

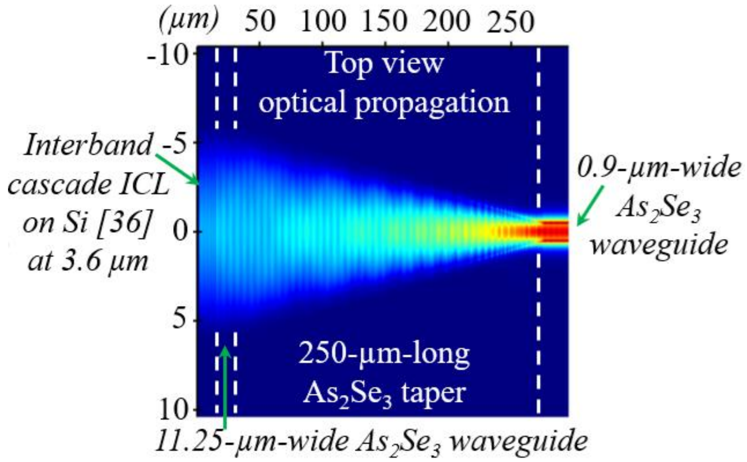

There is one power at specified mode, at the entrance of the 11.25 μm wide As_2Se_3 waveguide

There is one power at specified mode, at the exit of the laser

There is one power monitor at specified mode, after the mode source

Mode source

Employed material parameters for reported quantum cascade laser (QCL) on Si [64] at 4.8 um

Table 1. III-V Layers

Layers	Material	Thickness (nm)	Doping (cm-3)
Substrate	InP	–	–
Etch stop	InGaAs	50	–
Top contact	*n*-InP	1500	5×10^{18}
Top clad	*n*-InP	50	5×10^{17}
Top clad	*n*-InP	50	1×10^{17}
Top clad	*n*-InP	2450	2×10^{16}
Transition	*n*-InGaAs/InAlAs	50	1×10^{17}
Active core	*n*-QC structure	1510	Variable
Transition	*n*-InGaAs/InAlAs	50	1×10^{17}
Bottom clad	*n*-InP	50	1×10^{17}
Bottom clad	*n*-InP	50	5×10^{17}
Bottom contact	*n*-InP	200	1×10^{18}
Bonding SL	*n*-InP	7.5	1×10^{18}
Bonding SL	*n*-InGaAs	7.5	1×10^{18}
Bonding SL	*n*-InP	7.5	1×10^{18}
Bonding SL	*n*-InGaAs	7.5	1×10^{18}
Bonding layer	*n*-InP	10	1×10^{18}
Capping layer	*n*-InGaAs	200	1×10^{18}

Material	Refractive index (real)	Ref.
InAs	3.5498+i0.015623	Adachi 1989 (ref)
AlAs	3.1659+i0.020259	10.1088/0268-1242/17/11/310
GaAs	3.3265+i0.0018863	Adachi 1989 (ref)
InP		
InAs	3.4702+i0.0010524	Adachi 1989 (ref)
QCL		https://doi.org/10.1063/1.2770768
$Ga_{0.331}In_{0.669}As$	3.4759+i0.0111	0.331*GaAs+0.669*InAs
$Al_{0.638}In_{0.362}As$	3.3049+i0.0186	0.638*AlAs+0.362*InAs
InGaAs	3.4381+i0.0087	0.5*InAs+0.5*GaAs

Details on the treatment of the active laser regions are on the next slide.

Employed material parameters for reported quantum cascade laser (QCL) on Si [64] at 4.8 um

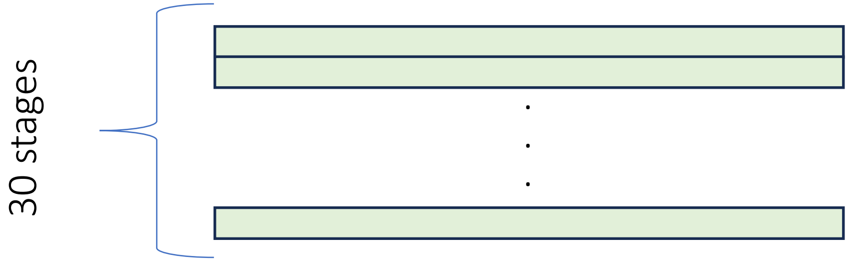

QCL layer has 30 stages

1 stage = 1 injector region / 1 active region
1 injector region = seven pairs of GaInAs/AlInAs
1 active region = four pairs of GaInAs/AlInAs

QCL structure

1. Injector region

Seven $Ga_{0.331}In_{0.669}As/Al_{0.638}In_{0.362}As$ pairs

(2.8/ 1.7), (2.5/ 1.8), (2.2/ 1.9), (2.1/ 2.1), (2.0/ 2.1), (1.8/ 2.7), and (1.8/ 3.8) [$Ga_{0.331}In_{0.669}As$ = 15.2 nm, and $Al_{0.638}In_{0.362}As$ = 16.1 nm]

2. active region

Four $Ga_{0.331}In_{0.669}As/Al_{0.638}In_{0.362}As$ pairs

(1.2/ 1.3), (4.3/ 1.3), (3.8/ 1.4), and (3.6/ 2.2) [$Ga_{0.331}In_{0.669}As$ = 12.9 nm and $Al_{0.638}In_{0.362}As$ = 6.2 nm]

QCL structure

30-stage Injector/active regions

Simulation structures and monitor positions used for verification along the optical part for the integration between quantum cascade laser (QCL) on Si [64] at 4.8 um

(from the external laser chip at 4.8 µm directly to the 0.4 µm wide As_2Se_3 waveguide without a taper)

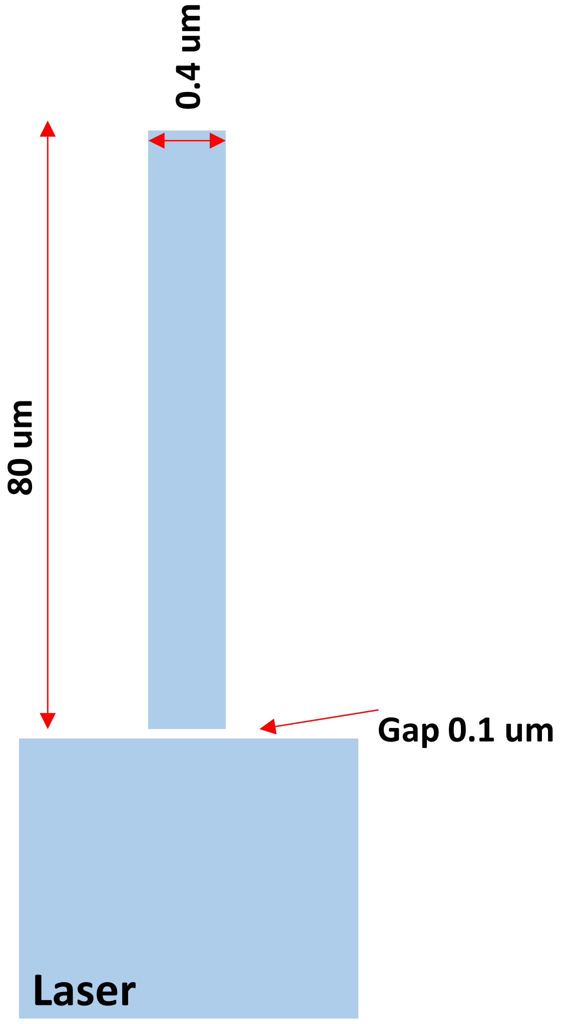

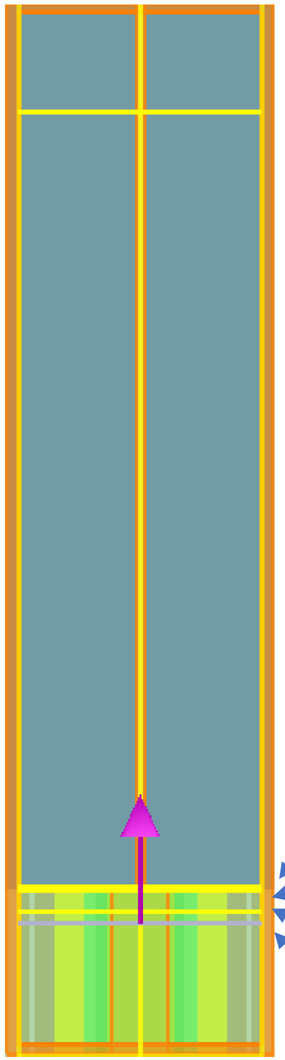

Power monitor at specified mode at the 0.4 µm wide As_2Se_3 waveguide for the reported transmitted optical power in the desired fundamental sensing mode at the As_2Se_3 waveguide with respect to the power from the input.

There is one power at specified mode, at the entrance of the 4 µm wide As_2Se_3 waveguide

There is one power at specified mode, at the exit of the laser

There is one power monitor at specified mode, after the mode source

Mode source

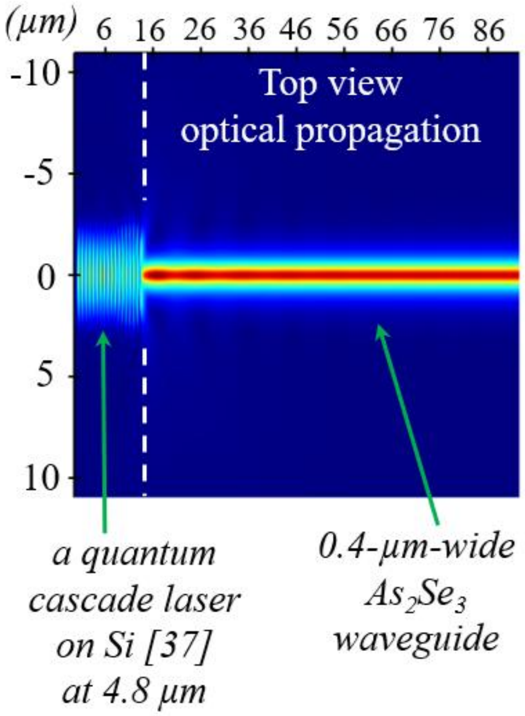

Employed material parameters for the reported DFB-QCL laser on Si [65] at 7.4 um

DFB cascade laser (Ref)
Operated at a **wavelength of 7.4 um,**
25 periods,
The width of the waveguide core is 10 um, Side walls is coated with SiN.

Material	Refractive index (real)	Ref.
GaAs	3.3265+i0.0018863	Adachi 1989 (ref)
InAs	3.4702+i0.0010524	Adachi 1989 (ref)
AlAs	2.831	Palik (ref)
$Ga_{0.47}In_{0.53}As$	3.403	0.47*GaAs+0.53*InAs
$Ga_{0.32}\ In_{0.678}As$	3.417	0.32*GaAs+0.678*InAs
$Al_{0.635}In_{0.365}As$	3.064	0.635*AlAs+0.365*InAs
InP, SiN		Lumerical database
Injector region	(3.417*15.75+3.064*17)/(15.75+17) = ***3.234***	
Active region	(3.417*13.25+3.064*6.3)/(13.25+6.3) = ***3.303***	
25-period QCL	(***3.234****32.75+***3.303****19.55)/(32.75+19.55) = 3.26	

Details on the treatment of the active laser regions are on the next slide.

Employed material parameters for the reported DFB-QCL laser on Si [65] at 7.4 um

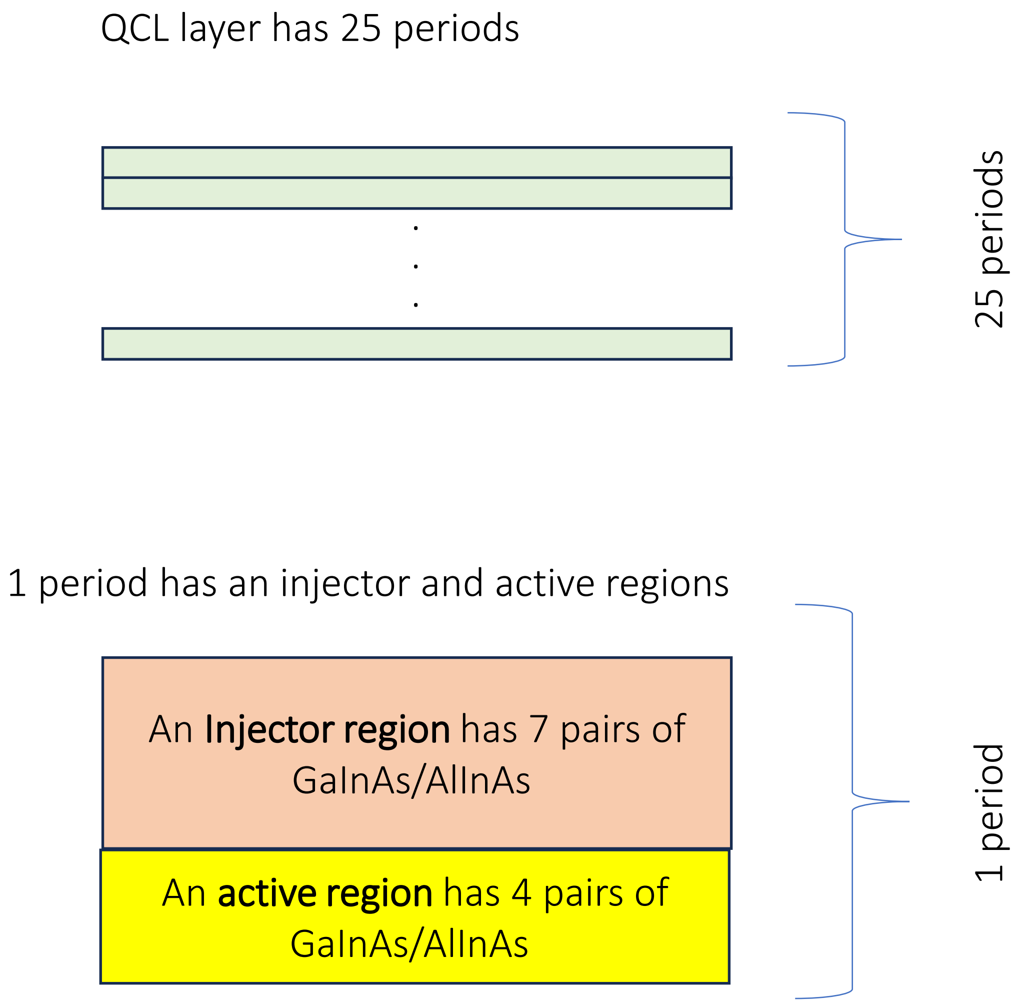

(nm)	Ga0.32 In0.678As	Al0.635In0.365As
Injector region	2.88	1.8
	2.58	1.9
	2.29	2
	2.19	2.2
	2.09	2.2
	1.86	2.9
	1.86	4
**Total thickness**	15.75	17
Active region	1.26	1.3
	4.4	1.3
	3.87	1.4
	3.72	2.3
**Total thickness**	13.25	6.3

Simulation structures and monitor positions used for verification along the optical part for the integration between the reported DFB-QCL laser on Si [65] at 7.4 um

(from the external laser chip at 7.4 µm to the 8.5 µm wide As_2Se_3 waveguide via a 250 µm long taper section to the 1 µm wide As_2Se_3 waveguide)

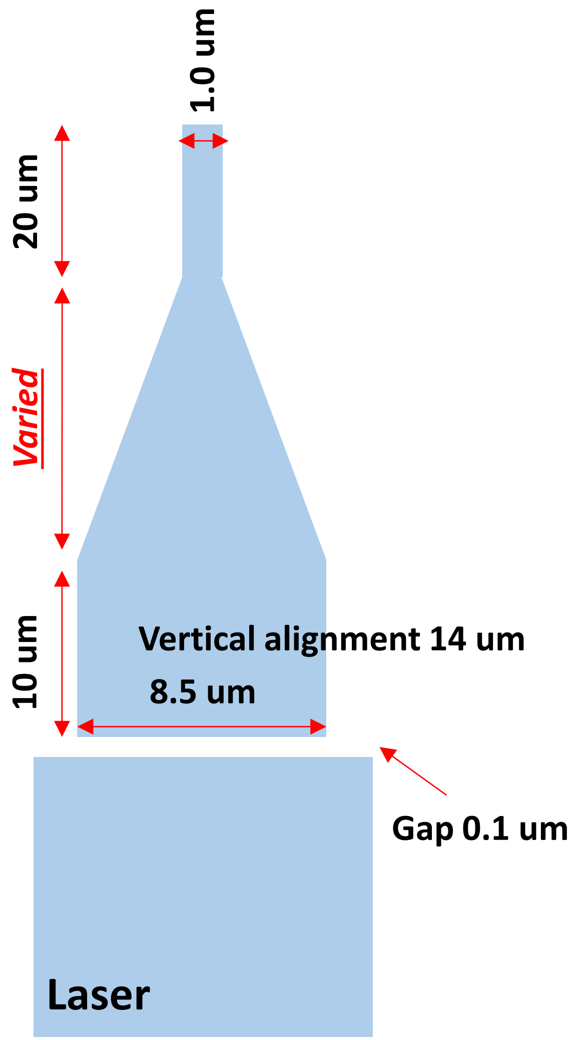

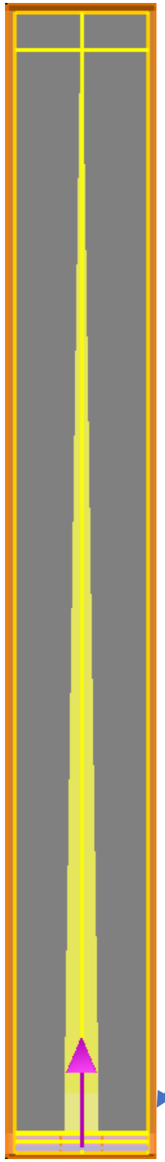

Power monitor at specified mode at the 1 µm wide As_2Se_3 waveguide for the reported transmitted optical power in the desired fundamental sensing mode at the As_2Se_3 waveguide with respect to the power from the input.

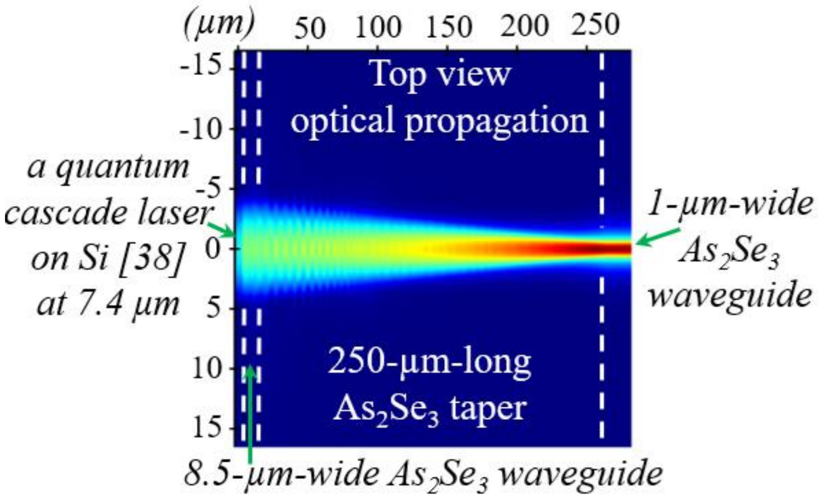

There is one power at specified mode, at the entrance of the 8.5 µm wide As_2Se_3 waveguide

There is one power at specified mode, at the exit of the laser

There is one power monitor at specified mode, after the mode source

Mode source

As_2Se_3

宁波大学 NINGBO UNIVERSITY

红外材料及器件实验室

Laboratory of Infrared Materials and Devices

NBU-IR5 （As_2Se_3）

Physical Data and Typical Characteristics

Mechanical

Density	g/cm³	4.63±0.01g/cm³
Young's modulus	GPa	18.3 Gpa
Rupture Modulus	MPa	17 Mpa

Thermal

Thermal change dn/dT	40×10^{-6} K^{-1} (10.6μm)
Expansion	20.6×10^{-6} K^{-1}
Specific Heat	0.36 J/gK
Thermal Conductivity	0.24 W/mK
Transition Temperature.	182 ℃

Optical

Wavelength (μm)	Index Of Refraction	Transmission (293K) %
2	2.8219	
3	2.8034	
4	2.7964	
5	2.7926	
6	2.7898	
7	2.7873	
8	2.7850	
9	2.7823	
10	2.7795	
11	2.7766	
12	2.7737	

**Formats**	Ingots<br>Disks
**Sizes**	Diameters up to 100 mm<br>Length of ingot up to 80 mm

M. Pi, C. Zheng, R. Bi, H. Zhao, L. Liang, Y. Zhang, Y. Wang, F. K. Tittel, "Design of a mid-infrared suspended chalcogenide/silica-on-silicon slot waveguide spectroscopic gas sensor with enhanced light-gas interaction effect," Sensors & Actu-ators: B. Chemical 297, 126732 (2019)

http://www.ir-glass.com/usr/uploads/2018/02/1392963412.pdf

Irving H. Malitson, "A Redetermination of Some Optical Properties of Calcium Fluoride," Appl. Opt. 2, 1103-1107 (1963)

As2Se3 (3.6) = 2.7992

As2Se3 (4.8) = 2.79336

As2Se3 (7.4) = 2.7864

CaF_2

CaF2	
wavelength(um)	refractive index
1	1.429
2	1.424
3	1.418
4	1.410
5	1.400
6	1.386
7	1.371
8	1.349

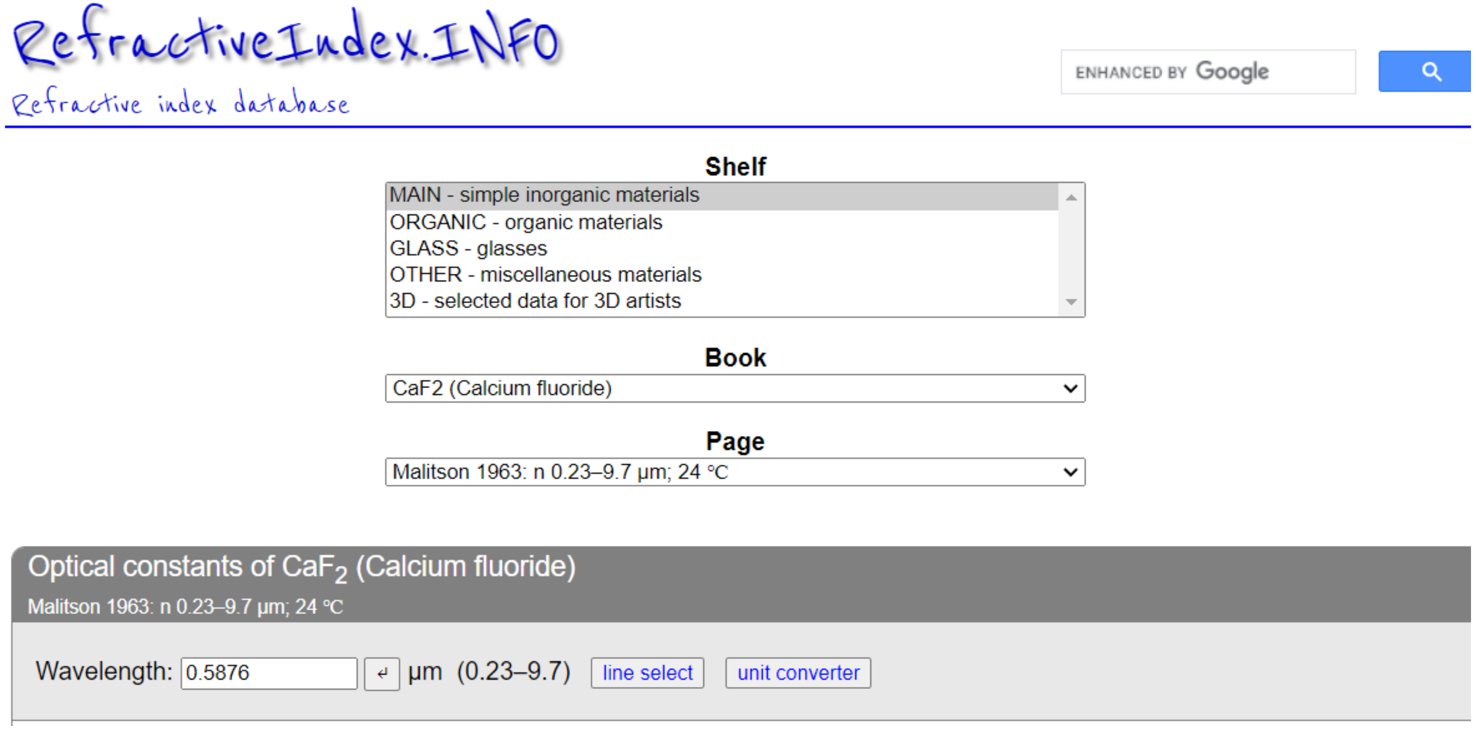

CaF2 (3.6) = 1.4132 (Interpolation)
CaF2 (4.8) = 1.4013 (Interpolation)
CaF2 (7.4) = 1.3619 (Interpolation)

Sidewall-scattering analysis

Approximate loss for

- 0.9 μm wide As_2Se_3 waveguide at 3.6 μm
- 0.4 μm wide As_2Se_3 waveguide at 4.8 μm
- 1 μm wide As_2Se_3 waveguide at 7.4 μm

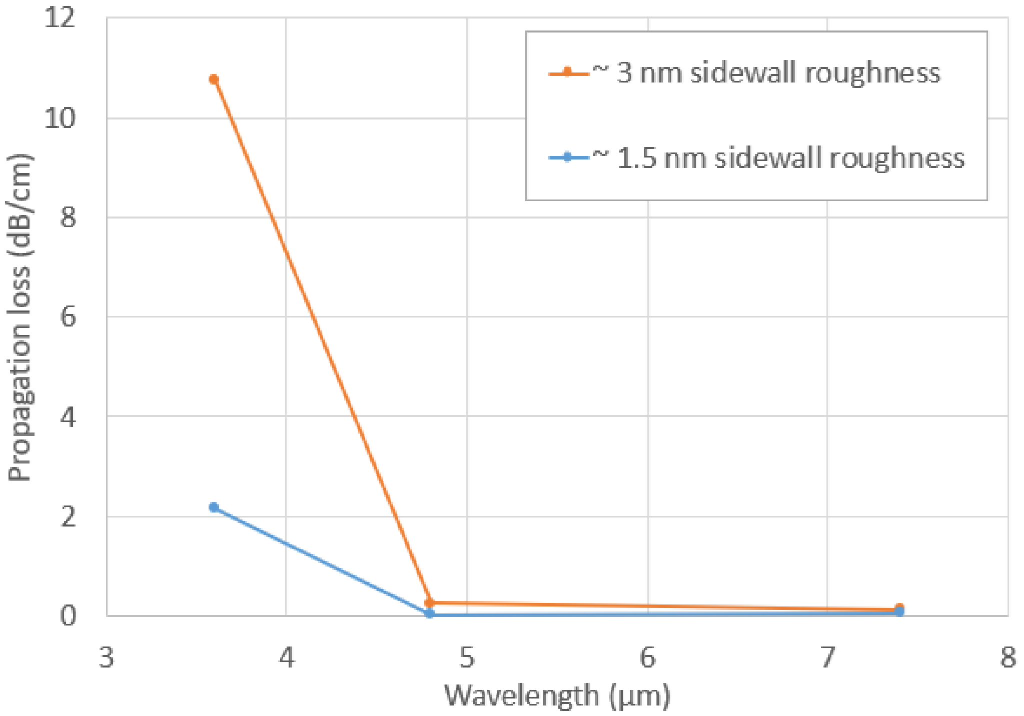

The employed correlation length is 400 nm. For lithographically defined chalcogenide waveguides, roughness is generally transferred from the resist edge [J. Hu et al.]; moreover, the value is comparable to 1/β in the 3–8 μm range, representing a pessimistic condition for sidewall scattering [F. P. Payne et al.] As expected, the sidewall-scattering loss is significantly larger at 3.6 μm (quasi-TE light) than that at 4.8 μm and 7.4 μm (quasi-TM light.)

- J. Hu, N.-N. Feng, N. Carlie, L. Petit, A. Agarwal, K. Richardson, and L. Kimerling, "Optical loss reduction in high-index-contrast chalcogenide glass waveguides via thermal reflow," *Optics Express* 18, 1469–1478 (2010).
- F. P. Payne and J. P. R. Lacey, "A theoretical analysis of scattering loss from planar optical waveguides," *Optical and Quantum Electronics* 26, 977–986 (1994).
